# Perinatal Health Care Among Climate Migrant Women: Protocol for a Scoping Review

**DOI:** 10.2196/84176

**Published:** 2026-02-17

**Authors:** Giti Khalaj, James Plaisimond, Thierno Diallo, Lisa Merry, Steven Ouellet, Mohamed Ali Ag Ahmed, Lara Maillet, Samira Amil, Marielle M'bangha, Erika Adriana Corona, Marie-Pierre Gagnon

**Affiliations:** 1 Faculty of Nursing, Université Laval, 1050 Av. de la Médecine, Québec, QC, G1V 0A6, Canada, 1 418 656 2131 ext 407576; 2 CHU de Québec – Université Laval Research Center, Québec, QC, Canada; 3 VITAM – Centre for Research in Sustainable Health, Québec, QC, Canada; 4 Faculty of Nursing, Université de Montréal, Montréal, QC, Canada; 5 SHERPA University Institute, Montréal, QC, Canada; 6 Administration publique et gestion des services de santé, École des hautes études publiques (HEP), Université de Moncton, Moncton, NB, Canada; 7 Department of Health Management, Evaluation and Policy, Université de Montréal, Montréal, QC, Canada; 8 École nationale d'administration publique, Montréal, QC, Canada; 9 School of Nutrition, Université Laval, Québec, QC, Canada; 10 Service de référence en périnatalité pour les femmes immigrantes de Québec, Québec, QC, Canada

**Keywords:** climate change, migration, women, maternal health, perinatal health care, prenatal period

## Abstract

**Background:**

Climate change–induced international migration has the potential to negatively impact the health and well-being of displaced populations. Pregnancy often serves as a point of entry into the health care system for migrant women; however, these women often face reduced access to maternal health care services compared to nonmigrants. In the context of climate-related international migration, these disparities may be further exacerbated, increasing the risk of maternal morbidity and adverse perinatal outcomes. While the intersections between climate change, migration, and health are increasingly acknowledged, literature specifically focused on international climate-related migrant women—particularly during the perinatal period—remains limited and dispersed. Thus, there is a growing need for research and synthesized data on climate change, population movements, and the perinatal health care needs of childbearing women.

**Objective:**

The aim of this study is to examine and describe the scope and nature of available evidence on maternal health and care for international climate-related migrant women, from pregnancy through the postpartum period.

**Methods:**

We will conduct a scoping review following the Joanna Briggs Institute methodology. A tailored search strategy using key terms related to climate change, migration, women, and perinatal health care will be applied to four databases—Embase, CINAHL, PsycINFO, and Ovid MEDLINE—without restriction on publication date. Relevant gray literature sources will also be searched and considered for inclusion. Only literature published in English, French, Portuguese, or Spanish will be included. Two reviewers will independently screen full-text records based on predefined inclusion criteria and extract the relevant data.

**Results:**

A total of 741 studies were screened from 1113 records. Results summarizing perinatal health outcomes and needs, care experiences, barriers faced by international climate-related migrant women, and recommendations will be reported using the PRISMA (Preferred Reporting Items for Systematic Reviews and Meta-Analyses) 2020 flow diagram. We anticipate finalizing the manuscript for this work in 2026.

**Conclusions:**

Considering vulnerability factors related to migration status is essential to improving access to integrated perinatal health care and reducing health inequities among immigrant women. This review will provide valuable insights to tailor interventions to the social and cultural needs of climate-affected migrant women during the perinatal period.

## Introduction

### Background

Climate change, as defined by the Intergovernmental Panel on Climate Change [1], refers to a change in the state of the climate that can be identified (eg, using statistical tests) by changes in the mean and/or the variability of its properties and that persists for an extended period, typically decades or longer. It refers to any change in climate over time, whether due to natural variability or as a result of human activity. Slow-onset climate processes refer to gradual environmental changes caused by climate change, such as sea-level rise, desertification, salinization, land degradation, biodiversity loss, glacial retreat, and changing rainfall patterns [2]. Unlike sudden-onset events, these processes unfold over longer time frames and can progressively undermine the viability of livelihoods, leading to delayed or protracted migration decisions.

Human migration in response to ecological changes has occurred since the origin of our species [3]. However, the current pressures that climate change exerts on human mobility are relatively recent and continue to intensify [4]. Climate change is increasingly understood as a “threat multiplier” because of its interaction with other economic, political, and social factors—exacerbating existing socioeconomic and political vulnerabilities and drivers of migration [5,6]. According to several projections, climate change is expected to substantially increase the scale of population displacement in the coming decades [7-11].

Climate migration is defined by the International Organization of Migration (IOM) as the movement of a person or groups of persons who, predominantly for reasons of sudden or progressive change in the environment due to climate change, are obliged to leave their habitual place of residence or choose to do so, either temporarily or permanently, within a state or across an international border [12]. These environmental changes can be sudden-onset events, such as floods or hurricanes, or slow-onset processes, such as prolonged droughts or sea-level rise, which gradually render areas uninhabitable [2]. For the purposes of this review, we will adopt this IOM definition of climate migration to guide study selection and screening.

Despite the growing importance of this phenomenon, global data remain fragmented and underresearched, particularly concerning migrations related to climate changes. Currently, there is no internationally recognized legal framework or formal immigration status for individuals displaced by climate change, which makes it nearly impossible to accurately estimate the prevalence of climate-related migration. This limitation is further complicated by the fact that climate-related factors often intersect with economic, political, and social drivers of migration, making causal attribution complex [13,14]. However, the increasing number of internal displacements triggered by sudden-onset climate events in recent years, alongside sustained international migration from countries severely affected by slow-onset climate changes, suggests that climate-related migration is steadily rising [1,15].

While migration is increasingly recognized for its adaptive potential in the context of climate change, and research on the health impacts of climate change is expanding, the specific health implications of climate-related migration remain underexplored. Climate-induced migration can have serious consequences for the health of migrated individuals, particularly in contexts of forced migration. The 2015 Paris Agreement acknowledges that women are disproportionately affected by climate change [16], suggesting that women’s health, especially during the perinatal period, is also disproportionately impacted on a global scale. Emerging evidence indicates that climate change can affect childbearing health through increased exposure to heat stress, air pollution, food and water insecurity, and disruptions to health care systems. These conditions are associated with heightened risks of adverse maternal and neonatal outcomes, such as preterm birth, low birth weight, stillbirth, and complications during labor [17,18].

Pregnancy often serves as a critical entry point into the health care system for migrant women [19]. Yet, migrant women are generally less likely than nonmigrant women to access adequate maternal health care services [19-22]. A systematic review by Heaman et al [23] reported that migrant women in Western industrialized countries are more likely to receive inadequate prenatal care, begin care later in pregnancy, and have fewer than the recommended number of prenatal visits. Childbearing migrant women—including those who are internally displaced, are living in refugee camps, or have crossed international borders—face increased health risks throughout the migration process. These groups often encounter multiple vulnerabilities, such as unstable housing, food insecurity, trauma, and limited access to culturally appropriate health care [24-27]. They are at greater risk of maternal morbidity and mortality and are less likely to access health care, particularly in humanitarian or low-resource contexts [28,29]. Given these intersecting vulnerabilities and the additional strain climate change places on health systems and migration patterns, women experiencing climate-related migration may face compounded risks and heightened barriers to perinatal care. Most studies have focused on women remaining within disaster-affected regions. As such, the perinatal risks associated with climate migration can only be postulated by extrapolating from existing evidence on the effects of migration and internal displacement on pregnancy. Although most migration driven by climate change tends to occur within national borders, some people are also compelled to cross international boundaries. However, comprehensive global data—like movements linked to slow-onset climate processes such as sea-level rise or land degradation—remain scarce [30].

While some reviews have addressed aspects of climate-related migration and perinatal or sexual and reproductive health [31,32], there remains a need for a focused scoping review that specifically maps evidence on the perinatal health and care experiences of international climate-related migrant women. Due to the limited evidence available on migration specifically linked to climate processes, a scoping review is warranted to map the existing evidence on the perinatal health and care experiences of different groups of international migrant women affected by climate change, including those affected by both sudden- and slow-onset climate processes, in order to inform practice and policy, identify knowledge gaps, and guide future research priorities aimed at improving maternal and newborn health outcomes in the context of a changing climate. Specifically, it aims to examine and describe the scope and nature of available evidence on maternal health and care for international climate-related migrant women, from pregnancy through the postpartum period.

### Review Question

What is known about the perinatal health and care of migrant women who have experienced climate-related migration?

### Subquestions

There are two subquestions this review aims to answer:

1. How do climate-related migrant women perceive perinatal health care services in host countries?
2. What barriers and challenges do they face in accessing perinatal health care services*?*

## Methods

### Overview

This scoping review will be conducted in accordance with the Joanna Briggs Institute (JBI) methodology for scoping reviews [33]. This framework involves a systematic process that includes the identification of the research questions, the comprehensive search for relevant literature, the selection of eligible literature, the extraction and charting of data, synthesis of the findings, and, where applicable, stakeholder consultation. To ensure methodological transparency and reproducibility, the review will adhere to the PRISMA-ScR (Preferred Reporting Items for Systematic Reviews and Meta-Analyses extension for Scoping Reviews) checklist and explanation [34]. This protocol will also be registered on the Open Science Framework to enhance transparency and facilitate access.

### Inclusion Criteria

The inclusion will align with the JBI framework including population, concept, and context (PCC) [35]. Articles addressing the perinatal health care received by climate-related migrant women during prepartum and postpartum will be included in our review.

We will include all types of evidence matching the PCC (population, concept, context) criteria.

### Population

We will include literature focusing on international climate-related migrant women during the perinatal period. During the screening process, studies will be included if participants are explicitly identified as climate-related migrants, defined as women who, in the perinatal period, predominantly for reasons of sudden or progressive environmental changes due to climate change (eg, floods, droughts, extreme heat, temperature anomalies, cyclones), are obliged to leave their habitual place of residence or choose to do so, either temporarily or permanently, within a state or across an international border.

### Concept

The concept under investigation is perinatal health, which encompasses maternal health (both physical and mental), access to and utilization of health care services, perceptions of care, and barriers and facilitators encountered.

### Context

We will include literature set in health care or community settings in host countries, including both high-income countries and low- and middle-income countries.

### Types of Sources

This scoping review will consider peer-reviewed research using qualitative, quantitative, or mixed methods designs, as well as existing reviews (such as systematic reviews, scoping reviews, and critical literature reviews). Relevant gray literature will also be included to ensure a comprehensive overview of the topic. This includes targeted sources such as the World Health Organization, the IOM, the World Bank, the United Nations High Commissioner for Refugees, the Internal Displacement Monitoring Center, other United Nations agencies, government and nongovernmental organization reports, and other policy documents.

### Search Strategy

A scoping review of studies reporting health outcomes and health care among climate-related migrant women, specifically concerning pregnancy and postpartum, will be conducted. A comprehensive literature search will be carried out across four electronic databases—Embase, CINAHL, PsycINFO, and Ovid MEDLINE—to identify relevant articles published in English, French, Portuguese, or Spanish (languages spoken by team members) with no restrictions on publication date. These databases will be selected based on their relevance to the research question and their comprehensive coverage of health, social science, and psychological literature coverage. The search strategy, which will incorporate both keywords and subject headings, will be developed in Ovid MEDLINE and then adapted for use in other databases. The search will be structured around main concepts: climate change, migration, women, and perinatal health care. Within each concept, related keywords and controlled vocabulary will be combined using the Boolean operator OR. The concepts themselves will then be combined using AND to ensure the retrieval of relevant and focused results. To supplement the electronic database search, backward and forward citation tracking of relevant publications will be conducted. Additional searches will also be performed using Semantic Scholar, leveraging tools such as Elicit and Consensus. Finally, targeted searches will be carried out using Google Scholar, where only the first 100 results will be assessed.

### Study/Source of Evidence Selection

Following the search, all identified citations will be collated and uploaded into Covidence (Veritas Health Innovation) [36], and duplicates will be removed. Following a pilot test, titles and abstracts will be screened by two independent reviewers for assessment against the inclusion criteria for the review. Two independent reviewers will assess the full text of selected citations in detail against the inclusion criteria. Reasons for the exclusion of sources of evidence at the full-text review stage that do not meet the inclusion criteria will be recorded and reported in the scoping review. Any disagreements between the reviewers at each stage of the selection process will be resolved through discussion or, if necessary, by consultation with an additional reviewer. The results of the search and the study inclusion process will be reported in full in the final scoping review and presented in a PRISMA (Preferred Reporting Items for Systematic Reviews and Meta-Analyses) 2020 flow diagram [37].

### Data Extraction

Data will be extracted from papers included in the scoping review by two independent reviewers using a data extraction tool developed by the research team in accordance with JBI guidance [38]. The draft data extraction tool will be piloted on a subset of studies to be included in the review to assess its feasibility. The finalized tool will be reported in the scoping review manuscript. The data extracted will include specific details about the descriptive data (title, year, authors, type of study), population (sample size; key characteristics; type of migrant—international migration, either forced or voluntary; source country; length of time—temporary or permanent; type of climate event), concept (perinatal health and care outcomes), context (country; health care or community settings), study/review design and methodology, and key findings relevant to the review questions. The quality of the included studies will be assessed using the Mixed Methods Appraisal Tool [39].

### Data Analysis and Presentation

Data extracted from the included sources will be analyzed using both quantitative and qualitative methods, depending on the type of data. We will first perform a descriptive analysis to summarize characteristics such as publication year, country of origin, study design, population, and type of evidence. This will include frequency counts and tabulations to map the distribution of the literature.

For qualitative data, a thematic analysis will be conducted. Two independent reviewers will code the extracted data using a structured spreadsheet in Microsoft Excel. Initial codes will be developed inductively, then refined iteratively through discussion and consensus. Discrepancies will be resolved by a third reviewer if necessary. Themes will then be systematically mapped to the review’s PCC elements to ensure alignment with the research questions, in accordance with the JBI recommendations [38].

A narrative synthesis will accompany the findings to contextualize and interpret the data in relation to the review’s objectives. Where appropriate, visual representations such as charts or thematic maps will be used to illustrate the key findings. All methods of data extraction and analysis will be guided by the JBI methodology for scoping reviews [33].

### Ethical Considerations

This scoping review does not involve the collection of primary data. Ethics approval was obtained from the *Center intégré universitaire de santé et de services sociaux de la Capitale-Nationale*, through the *Comité d’Éthique de la Recherche Sectoriel* evaluator for Population Health and Primary Care (approval number 2025‐3314). The project underwent both scientific and ethical review and received favorable evaluations, as well as suitability approval from the managers of the participating facilities and clinical departments.

## Results

A preliminary search applying the key terms across all 4 databases yielded 1113 records. After duplicates were removed using the Covidence platform, 741 records were retained for further screening. Next, titles and abstracts will be reviewed, followed by a detailed assessment of full-text articles by two reviewers. A preliminary PRISMA-ScR flow diagram outlining the planned selection process is presented using the PRISMA 2020 flow diagram [37]. The PRISMA diagram can be found in Multimedia Appendix 1. Numbers marked as TBD (to be determined) will be updated once the review is completed. We anticipate finalizing the manuscript in 2026.

## Discussion

### Anticipated Findings

This scoping review is expected to provide an initial mapping of the evidence describing the perinatal health needs, care experiences, and barriers faced by international climate-related migrant women. Based on preliminary knowledge and the anticipated synthesis of existing literature, we expect to identify recurrent challenges such as limited access to timely and culturally appropriate perinatal care, gaps in service coordination, and broader systemic vulnerabilities linked to both migration status and climate-related displacement. It is also anticipated that the review will highlight substantial gaps in research, including the limited documentation of climate-related migration as a determinant of perinatal health and the fragmented nature of available studies across disciplines.

### Comparison to Prior Work

Two previous reviews [31,32] have explored climate-related migration in relation to sexual or reproductive health, and none have focused specifically on perinatal health among international climate-related migrants. By narrowing the scope to this population, our review aims to offer a more granular understanding of how climate-related drivers intersect with migration pathways and perinatal health outcomes. This work is expected to contribute conceptual clarity by identifying inconsistencies in how climate-related migrants are defined and operationalized, while also bringing forward methodological gaps such as the absence of standardized indicators or the underreporting of migration drivers in maternal health research.

### Strengths

A key strength of this review is the use of the JBI methodology, which ensures rigor, transparency, and reproducibility throughout each stage of the review process. The comprehensive search strategy—spanning 4 multidisciplinary databases and incorporating gray literature—maximizes the likelihood of capturing diverse forms of evidence across countries and contexts. The multilingual expertise of the review team (English, French, Portuguese, and Spanish) is an additional strength, increasing the inclusiveness of the review and enhancing its ability to identify relevant literature from multiple regions.

### Limitations

Several limitations must be acknowledged. First, there is no universally accepted definition or legal status for climate-related migrants, and many studies do not explicitly identify this group, which may lead to underrepresentation or inconsistent terminology across the included evidence. Second, despite including 4 languages, literature published in other languages may remain inaccessible, potentially limiting the global representativeness of the findings. Third, given the expected diversity in study designs, contexts, and conceptual frameworks, heterogeneity across studies may limit comparability and hinder synthesis. Finally, as a scoping review, the study will not assess the quality of included evidence, and conclusions will be descriptive rather than evaluative. A preliminary quality assessment using the Mixed Methods Appraisal Tool will provide context, but the synthesis will remain descriptive.

### Future Directions

The anticipated findings will help identify conceptual, methodological, and empirical gaps that can guide future research. In particular, the review may highlight the need for (1) improved identification and documentation of climate-related migrants in health research; (2) standardized approaches for capturing climate-related drivers of migration in perinatal health studies; (3) longitudinal or mixed methods research examining the long-term perinatal outcomes of climate-related displacement; and (4) studies exploring culturally adapted models of perinatal care that address both social and environmental determinants of health. Additionally, the review may point to the need for coordinated policy responses that integrate climate adaptation strategies with migrant-sensitive maternal health services.

### Dissemination Plan

In alignment with *JMIR Research Protocols* guidelines, the findings of this review will be disseminated through publication in an open-access journal; presentations at international conferences on maternal health, migration, and climate change; and knowledge translation activities aimed at policymakers, community organizations, and health care providers. Summaries of results will also be shared through institutional networks and public-facing formats such as infographics, policy briefs, or webinars to maximize accessibility and impact.

This scoping review will provide an evidence-based overview of the perinatal health needs, challenges, and care experiences of international climate-related migrant women. By identifying research gaps and mapping key determinants, it will enhance understanding of the intersecting social and environmental factors affecting this population. The anticipated findings may inform the development of inclusive, culturally responsive, and equitable perinatal health care strategies, as well as guide future research and policy initiatives.

## Supplementary material

10.2196/84176 Multimedia Appendix 1 PRISMA flow diagram.

10.2196/84176 Peer Review Report 1 Peer review report by the Canadian Institutes of Health Research.

## Abbreviations

IOM: International Organization for Migration
JBI: Joanna Briggs Institute
PCC: population, concept, context
PRISMA: Preferred Reporting Items for Systematic Reviews and Meta-Analyses
PRISMA-ScR: Preferred Reporting Items for Systematic Reviews and Meta-Analyses Extension for Scoping Reviews
