# Supplementary figures and images for: Perinatal Health Care Among Climate Migrant Women: Protocol for a Scoping Review
Source: JMIR Res Protoc. 2026 Feb 17;15:e84176. doi: 10.2196/84176 (PMC12912655; doi:10.2196/84176)

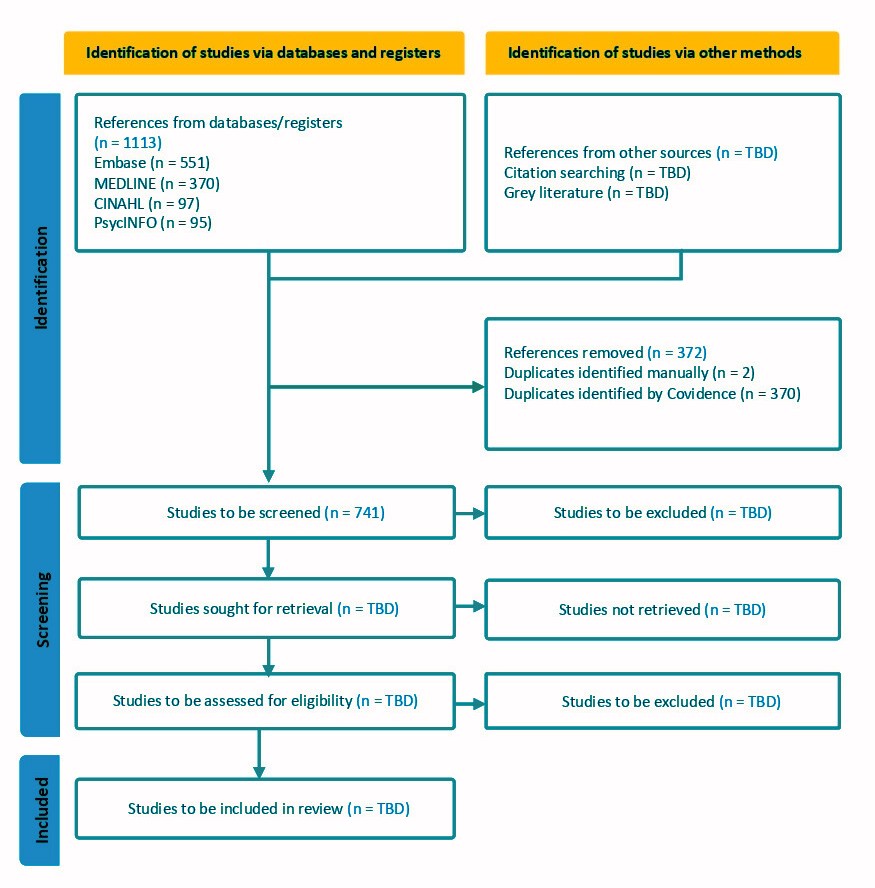

Supplement: Multimedia Appendix 1 [file resprot-v15-e84176-s001.jpg]
