# Supplementary material for: Perinatal Health Care Among Climate Migrant Women: Protocol for a Scoping Review
Source: JMIR Res Protoc. 2026 Feb 17;15:e84176. doi: 10.2196/84176 (PMC12912655; doi:10.2196/84176)
Supplement: Peer Review Report 1 [file resprot-v15-e84176-s002.pdf]

February/Février 20, 2025

DC0190GP

Canadian Institutes of Health Research / Instituts de recherche en santé du Canada

Notice of Decision / Avis de décision

Application Number/Numéro de la demande: 528380
Committee Code/Code du comité: CCR

Applicants/Candidats: Docteure Marie-Pierre Gagnon   Mrs. Marielle Mbangha

With/Avec: Monsieur M. Ag Ahmed   Professeur T. Diallo   Docteure L. Maillet   Ms. L. Merry

Institution paid/ Établissement payé: Université Laval

Title/Titre: Santé des familles immigrantes dans un contexte de justice climatique et de justice sociale (SI-JUSTE)

Primary Inst./ Inst. principal: Health Services and Policy Research / Services et politiques de la santé

Other Related Inst./ Autres inst. connexes: Gender and Health / Santé des femmes et des hommes; Population and Public Health / Santé publique et des populations; Human Development, Child and Youth Health / Développement et santé des enfants et des adolescents

Competition Outcome/Résultats du concours: Catalyst Grant: Community Based Research in Climate Change Priority Areas / SC: Rech. communautaire dans domaines prioritaires liés au changement climatique

September/Septembre 12, 2024

Number in competition/Nbre de demandes dans le concours: 69

Number approved in the competition / Nombre de demandes approuvées dans le concours: 42

Decision on your application/ Décision sur votre demande: Approved / Approuvée   Autre offre / Other offer

Average annual amount/ Montant annuel moyen: $125,000

Term/Durée: 1 **yrs/ans** 0 **months/mois**

Peer Review Committee Recommendation, for your information and use/ Recommandation du comité d'examen par les pairs, pour fins d'information et d'utilisation:

Committee/Comité: Community Based Research in Climate Change Priority Areas / Recherche communautaire dans les domaines prioritaires du changement climatique

Application rank within the competition/ Rang de la demande dans ce concours: 40

Percent Rank Within the Competition/ Rang en pourcentage au sein du concours: 57.97%

Rating/ Cote: 3.93

Recommended average annual amount/ Montant annuel moyen recommandé: $125,000

Additional Funding Opportunities/ Opportunités de financement additionnelles	Decision/ Décision	Total Funding Amount/Montant total du financement	Term/ Terme	Competition Code/Cote de concours	Application Number/ Numéro de la demande
Catalyst Grant: CBR in Climate Change Priority Area - Climate Justice/SC : RC dans domaines prioritaires liés au chang. climatique-Justice climatique	Approuvée/ Approved	$125,000	1 yr/an 0 mth/mois	202409RC3	538715

*** Applications receiving a score of less than 3.5 on any evaluation criteria will not be considered for Funding. / Les demandes qui ont reçu une note inférieure à 3.5 pour n'importe quel des critères d'évaluation ne sont pas admissibles.

This document is for information only. Official payment is stated on the CIHR Authorization for Funding.
Document à titre d'information seulement. Le paiement officiel est indiqué sur l'autorisation de financement des IRSC.

Canadian Institutes of Health Research
160 Elgin Street, 9th Floor
Address Locator 4809A
Ottawa, Ontario K1A 0W9

Instituts de recherche en santé du Canada
160, rue Elgin, 9e étage
Indice de l'adresse 4809A
Ottawa (Ontario) K1A 0W9

Institute of Aging
Institute of Cancer Research
Institute of Circulatory and Respiratory Health
Institute of Gender and Health
Institute of Genetics
Institute of Health Services and Policy Research
Institute of Human Development and Child and Youth Health
Institute of Indigenous Peoples' Health
Institute of Infection and Immunity
Institute of Musculoskeletal Health and Arthritis
Institute of Neurosciences, Mental Health and Addiction
Institute of Nutrition, Metabolism and Diabetes
Institute of Population and Public Health

Institut du vieillissement
Institut du cancer
Institut de la santé circulatoire et respiratoire
Institut de la santé des femmes et des hommes
Institut de génétique
Institut des services et des politiques de la santé
Institut du développement et de la santé des enfants et des adolescents
Institut de la santé des Autochtones
Institut des maladies infectieuses et immunitaires
Institut de l'appareil locomoteur et de l'arthrite
Institut des neurosciences, de la santé mentale et des toxicomanies
Institut de la nutrition, du métabolisme et du diabète
Institut de la santé publique et des populations

Le 20 février 2025

Docteure Marie-Pierre Gagnon
Faculté des sciences infirmières
Université Laval
Pavillon Ferdinand-Vandry
VND-1426
Québec, Québec G1V 0A6

Bonjour,

Au nom des Instituts de recherche en santé du Canada (IRSC) nous avons le plaisir de vous informer que la demande de subvention que vous avez présentée récemment au concours Subvention catalyseur : Recherche communautaire dans des domaines prioritaires liés au changement climatique qui s'intitule "Santé des familles immigrantes dans un contexte de justice climatique et de justice sociale (SI-JUSTE)" a été approuvée dans la classe Justice climatique. Cette subvention est financée par la Stratégie de recherche axée sur le patient du Canada.

Les documents relatifs à vos évaluations se trouvent sur RechercheNet. Veuillez noter que votre autorisation de financement vous sera envoyée par courriel. Étant donné que les IRSC n'avisent pas les co-candidats, nous vous prions de communiquer le résultat de cette demande aux personnes concernées ainsi qu'à leur établissement de recherche (s'il diffère du vôtre).

Pour toute question veuillez communiquer avec l'équipe de soutien des IRSC par courriel à support-soutien@cihr-irsc.gc.ca. Nous vous prions de ne pas communiquer avec les agents ou les membres du comité d'examen par les pairs.

Vous félicitant de votre succès à ce concours, nous vous prions d'agréer nos salutations distinguées.

Sincèrement,

Paula Kirton

Paula Kirton
Directrice générale, Conception et exécution des programmes

518799-202409RC3-CCR-538715-I013-99278-CCRA

CIHR IRSC
Discoveries for life / Découvertes pour la vie

Canada

Portefeuille des programmes de recherche

Canadian Institutes of Health Research
160 Elgin Street, 9th Floor
Address Locator 4809A
Ottawa, Ontario K1A 0W9

Instituts de recherche en santé du Canada
160, rue Elgin, 9e étage
Indice de l'adresse 4809A
Ottawa (Ontario) K1A 0W9

Institute of Aging
Institute of Cancer Research
Institute of Circulatory and Respiratory Health
Institute of Gender and Health
Institute of Genetics
Institute of Health Services and Policy Research
Institute of Human Development and Child and Youth Health
Institute of Indigenous Peoples' Health
Institute of Infection and Immunity
Institute of Musculoskeletal Health and Arthritis
Institute of Neurosciences, Mental Health and Addiction
Institute of Nutrition, Metabolism and Diabetes
Institute of Population and Public Health

Institut du vieillissement
Institut du cancer
Institut de la santé circulatoire et respiratoire
Institut de la santé des femmes et des hommes
Institut de génétique
Institut des services et des politiques de la santé
Institut du développement et de la santé des enfants et des adolescents
Institut de la santé des Autochtones
Institut des maladies infectieuses et immunitaires
Institut de l'appareil locomoteur et de l'arthrite
Institut des neurosciences, de la santé mentale et des toxicomanies
Institut de la nutrition, du métabolisme et du diabète
Institut de la santé publique et des populations

Le 20 février 2025

Docteure Marie-Pierre Gagnon
Faculté des sciences infirmières
Université Laval
Pavillon Ferdinand-Vandry
VND-1426
Québec, Québec G1V 0A6

Bonjour,

Au nom des Instituts de recherche en santé du Canada (IRSC), j'aimerais vous féliciter de votre succès au récent concours de financement des IRSC!

En tant que chercheurs du domaine de la santé, nous partageons un objectif commun : améliorer la santé et le bien-être des populations, au Canada et dans le monde. Par l'entremise des IRSC, le gouvernement du Canada offre un soutien essentiel aux chercheurs canadiens œuvrant dans tous les thèmes de la recherche en santé, qui sont étroitement liés, afin d'améliorer la santé de toute la population canadienne. Titulaire d'une subvention des IRSC, vous faites maintenant partie de cette entreprise.

L'évaluation de votre demande de subvention a été possible grâce aux pairs évaluateurs qui ont généreusement donné de leur temps pour soutenir le secteur canadien de la recherche en santé. En tant que titulaire de fonds des IRSC, vos connaissances et votre expertise sont fort précieuses. C'est pourquoi je vous encourage à participer à l'évaluation par les pairs et à envisager de devenir membre du Collège des évaluateurs, si vous ne l'êtes pas déjà, afin de soutenir ce processus aux IRSC. Veuillez consulter la page https://cihr.gc.ca/evaluer.html pour prendre connaissance des critères d'admissibilité et vous renseigner sur la façon d'évaluer les demandes aux IRSC. Les boursiers postdoctoraux et les stagiaires sont encouragés à explorer le cheminement des évaluateurs des IRSC (https://cihr-irsc.gc.ca/f/51666.html) afin d'avoir l'occasion de participer au processus d'évaluation et à des programmes de perfectionnement.

Aujourd'hui, l'activité scientifique est surveillée de près et il est plus important que jamais de veiller à ce que notre travail soit bien compris. Je vous demande donc de faire mention du financement des IRSC dans vos présentations et vos communications au sujet de vos recherches, et de continuer de faire valoir l'importance cruciale de la recherche pour la santé des Canadiennes et des Canadiens. Vous trouverez à l'adresse https://cihr-irsc.gc.ca/f/30789.html des moyens de reconnaître le soutien reçu et découvrirez comment l'équipe des Communications des IRSC peut vous aider à faire connaître votre recherche.

Encore une fois, je tiens à vous féliciter de cet exploit et à vous offrir mes meilleurs vœux de succès dans toutes vos entreprises. Je suivrai avec intérêt l'avancement de vos recherches.

Je vous prie de recevoir mes plus cordiales salutations.

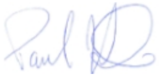

Paul C. Hébert, M.D., MHSc, FRCPC, MACSS
Président

518775-202409RC3-CCR-538715-I013-99278-CONGR

Canada
CIHR IRSC
Discoveries for life / Découvertes pour la vie

Review Type/Type d'évaluation: Committee Member 1/Membre de comité 1
Name of Applicant/Nom du chercheur: Gagnon, Marie-Pierre
Application No./Numéro de demande: 528380
Agency/Agence: CIHR/IRSC
Competition/Concours: 2024-09-12 Catalyst Grant: Community Based Research in Climate Change Priority Areas/Subvention catalyseur : Recherche communautaire dans domaines prioritaires liés au changement climatique
Committee/Comité: Community Based Research in Climate Change Priority Areas/Recherche communautaire dans les domaines prioritaires du changement climatique
Title/Titre: Santé des familles immigrantes dans un contexte de justice climatique et de justice sociale (SI-JUSTE)

Assessment/Évaluation:

Ce projet de recherche vise à explorer les défis rencontrés par les familles migrantes climatiques au Québec, en se concentrant sur leur adaptation à un nouvel environnement et l'accès aux ressources de santé. Il cherche à comprendre le processus d'adaptation, identifier les besoins en matière de santé, et développer des outils d'information adaptés pour des femmes immigrantes vivant un processus périnatal. Utilisant une approche de recherche action participative et l'ethnographie focalisée, le projet implique des entrevues auprès de mères immigrantes et des groupes de discussion avec des intervenants en santé. Les résultats attendus incluent la promotion de l'équité en santé et le développement d'outils pour améliorer l'intégration des familles migrantes climatiques au Québec.

Forces
- le projet répond à un besoin identifié par le partenaire communautaire, bonifiera son expertise en matière d' équité en santé des familles migrantes climatiques, et l'outillera pour mieux répondre aux besoins de ces familles en codéveloppant et en évaluant des solutions pour accroitre l'accessibilité aux services de santé pour les mères immigrantes et leurs familles
- Par la participation active des femmes immigrantes, la composition de l'équipe de recherche et la contribution du partenaire à la co-construction des connaissances, le projet aura une contribution importante à l'équité en santé des familles migrantes et comble d'importantes lacunes dans la littérature scientifique et grise en matière d'impact de la migration environnementale forcée sur la santé de nouveaux arrivants au Canada et au Québec
- La faisabilité est clairement démontrée tant par la synergie du partenariat; l'apport des compétences, des ressources et des expertises complémentaires et multidisciplinaires; et un budget équilibré qui prend en considération les besoins des participant.e.s (interprétation, services de garde, dédommagement) et le temps et l'expertise de la dirigeante communautaire

Faiblesses
Dans le cadre du parcours migratoire des participantes, nous invitons l'équipe à se pencher davantage/aussi sur les expériences vécues des femmes en matière de changements climatiques **à l'origine** de leur immigration forcée (et pas seulement sur leur expérience de transition) et d'en tenir compte dans l'analyse à partir d'une approche intersectionnelle (aléas, vulnérabilités, événement climatiques, statut socioéconomique, etc.). Cela a le potentiel de mieux circonscrire le contexte de migration pour cette population au Québec et au Canada.

Notions de sexe et de genre
Le projet vise spécifiquement à créer l'équité en santé pour les femmes immigrantes et leurs familles, ce qui montre une prise en compte claire du genre dès le départ. L'utilisation d'une approche de recherche action

**Review Type/Type d'évaluation:**	Committee Member 1/Membre de comité 1
**Name of Applicant/Nom du chercheur:**	Gagnon, Marie-Pierre
**Application No./Numéro de demande:**	528380
**Agency/Agence:**	CIHR/IRSC
**Competition/Concours:**	2024-09-12 Catalyst Grant: Community Based Research in Climate Change Priority Areas/Subvention catalyseur : Recherche communautaire dans domaines prioritaires liés au changement climatique
**Committee/Comité:**	Community Based Research in Climate Change Priority Areas/Recherche communautaire dans les domaines prioritaires du changement climatique
**Title/Titre:**	Santé des familles immigrantes dans un contexte de justice climatique et de justice sociale (SI-JUSTE)

Assessment/Évaluation:

participative et de méthodologies qualitatives (entretiens individuels et discussions de groupe) permet de recueillir des données détaillées sur les expériences spécifiques des femmes migrantes. Cela montre une volonté de comprendre en profondeur les besoins et les défis liés au genre. En impliquant les partenaires communautaires et les femmes migrantes dans le processus de recherche, le projet assure que les voix des femmes sont entendues et que les solutions développées sont adaptées à leurs besoins spécifiques. Ainsi, la prise en compte du sexe et du genre est une force de ce projet.

**Review Type/Type d'évaluation:**	Committee Member 2/Membre de comité 2
**Name of Applicant/Nom du chercheur:**	Gagnon, Marie-Pierre
**Application No./Numéro de demande:**	528380
**Agency/Agence:**	CIHR/IRSC
**Competition/Concours:**	2024-09-12 Catalyst Grant: Community Based Research in Climate Change Priority Areas/Subvention catalyseur : Recherche communautaire dans domaines prioritaires liés au changement climatique
**Committee/Comité:**	Community Based Research in Climate Change Priority Areas/Recherche communautaire dans les domaines prioritaires du changement climatique
**Title/Titre:**	Santé des familles immigrantes dans un contexte de justice climatique et de justice sociale (SI-JUSTE)

Assessment/Évaluation:

Résumé de la demande

Ce projet vise à explorer en profondeur le vécu et l'expérience de navigation et de l'utilisation des soins de santé – surtout périnataux – par les femmes migrantes ayant vécu un déplacement forcé par les conditions environnementales. Comme les migrants environnementaux n'ont pas nécessairement le vécu typiquement présenté par les demandeurs d'asile politique, ils sont dans une situation particulièrement précaire. Le projet est une collaboration surtout entre la FSI de l'Université Laval et un organisme communautaire, le SRPFIQ, qui offre un éventail d'informations, des ressources et des services liés à la santé périnatale. Plusieurs autres collaborateurs font partie de l'équipe. Le projet commence par une éthnographie focalisée du vécu de 8-10 femmes migrantes environnementales, suivi d'un groupe de discussion et d'un atelier de co-développement de prochaines démarches pour mieux répondre aux réalités et aux besoins de ces personnes.

1. Approche de recherche

Forces

Solide protocole de recherche communautaire par une équipe expérimentée et avec une histoire importante de collaboration. Cadre conceptuel et approche méthodologique solides, y compris l'analyse et l'atelier de co-construction. Projet faisable, grâce au SRPFIQ. Exploration d'une sous-population des personnes migrantes – les migrants environnementaux – qui est en croissance mais au sujet de laquelle les savoirs actuels sont limités. Sujet très pertinent au Québec actuellement. Le volet co-construction est une force particulière.

Faiblesses

La majeure faiblesse est que je ne suis pas convaincue que le projet tel que proposé pourra distinguer le vécu et les besoins de femmes migrantes environnementales vs d'autres femmes migrantes. Le lien avec les objectifs de ce concours est donc un peu faible, sans un volet comparatif. L'ethnographie focalisée basée que sur les entretiens semi-structurés (même approfondis) et un groupe de discussion me semble un peu mince comme ethnographie, bien que tout à fait acceptable comme recherche qualitative. Il n'est pas clair si une partie ou toutes les femmes ayant participé dans lesentrevues vont aussi participer dans l'atelier. Il aurait été pertinent d'explorer davantage le contexte plus large dans lequel se situent ces femmes migrantes ainsi que le SRPFIQ.

2. Candidats

Forces

Solides chercheur.e.s et solide collaboration de plusieurs années entre la CP et la directrice du SRPFIQ. Bonne complémentarité de formations et d'expériences

Faiblesses

L'expérience dans la recherche communautaire avec les réfugiés et surtout des réfugiés environnementaux (ou les politiques liées à l'accès aux soins pour les réfugiés environnementaux) n'est pas très bien reflétée dans les cv des chercheurs qui seront les plus responsables pour le déroulement de ce projet.

**Review Type/Type d'évaluation:**	Committee Member 2/Membre de comité 2
**Name of Applicant/Nom du chercheur:**	Gagnon, Marie-Pierre
**Application No./Numéro de demande:**	528380
**Agency/Agence:**	CIHR/IRSC
**Competition/Concours:**	2024-09-12 Catalyst Grant: Community Based Research in Climate Change Priority Areas/Subvention catalyseur : Recherche communautaire dans domaines prioritaires liés au changement climatique
**Committee/Comité:**	Community Based Research in Climate Change Priority Areas/Recherche communautaire dans les domaines prioritaires du changement climatique
**Title/Titre:**	Santé des familles immigrantes dans un contexte de justice climatique et de justice sociale (SI-JUSTE)

Assessment/Évaluation:

3. Impact de la recherche

Forces

Ce projet pourra avoir un fort impact sur les organismes participants, et contribuera à une attention accrue aux vécus et aux besoins des réfugiés environnementaux par les chercheurs et les associations professionnelles. Le projet fournira une solide base pour des futures recherches et interventions.

Faiblesses

Il n’est pas très évident que le projet aurait un impact au-delà des personnes et les organisations/équipes directement impliquées.

Recommandation budgétaire Budget approprié

**Review Type/Type d'évaluation:**	Committee Member 3/Membre de comité 3
**Name of Applicant/Nom du chercheur:**	Gagnon, Marie-Pierre
**Application No./Numéro de demande:**	528380
**Agency/Agence:**	CIHR/IRSC
**Competition/Concours:**	2024-09-12 Catalyst Grant: Community Based Research in Climate Change Priority Areas/Subvention catalyseur : Recherche communautaire dans domaines prioritaires liés au changement climatique
**Committee/Comité:**	Community Based Research in Climate Change Priority Areas/Recherche communautaire dans les domaines prioritaires du changement climatique
**Title/Titre:**	Santé des familles immigrantes dans un contexte de justice climatique et de justice sociale (SI-JUSTE)

Assessment/Évaluation:

Le projet semble bien structuré et présente un potentiel intéressant, avec une équipe qualifiée et un budget détaillé qui renforce la crédibilité du projet. Le projet présente un potentiel intéressant mais nécessite des clarifications et des ajustements pour être pleinement convaincant:

- Les critères de sélection des participants ne sont pas suffisamment clairs. Il est important de préciser comment vous allez identifier les personnes qui seront sollicitées et les raisons de ce choix.
- Le focus géographique semble nécessaire pour cibler les problématiques environnementales spécifiques. Quels sont les critères qui permettront de différencier les refus liés à l'environnement des autres ?

- Le projet reconnaît les limites en matière de littératie de certains participants. Il est essentiel de détailler les mesures qui seront mises en place pour assurer une participation équitable de tous.

- Le projet met l'accent sur le partage des connaissances, mais il manque d'informations sur les modalités de restitution aux participants. Comment les résultats de la recherche seront-ils communiqués ? Quelles actions seront mises en place pour valoriser leur contribution ?

- Le nombre de femmes participantes (8-10) semble faible. Il serait intéressant de justifier ce chiffre et d'expliquer comment il a été déterminé.

Review Type/Type d'évaluation: Committee Member 4/Membre de comité 4
Name of Applicant/Nom du chercheur: Gagnon, Marie-Pierre
Application No./Numéro de demande: 528380
Agency/Agence: CIHR/IRSC
Competition/Concours: 2024-09-12 Catalyst Grant: Community Based Research in Climate Change Priority Areas/Subvention catalyseur : Recherche communautaire dans domaines prioritaires liés au changement climatique
Committee/Comité: Community Based Research in Climate Change Priority Areas/Recherche communautaire dans les domaines prioritaires du changement climatique
Title/Titre: Santé des familles immigrantes dans un contexte de justice climatique et de justice sociale (SI-JUSTE)

Assessment/Évaluation:

Résumé de la demande

Cette demande vise à comprendre les effets du processus de migration environnementale sur des femmes dans la période périnatale dans la région de Québec. Elle s'articule selon 3 objectifs visant à analyser le processus d'adaptation des familles migrantes, identifier leurs besoins en termes d'accès aux ressources de santé et codévelopper des outils d'information sur les ressources de santé adapté. L'implantation et l' évaluation des outils fera partie d'un projet subséquent.

1. Approche de recherche

Forces

Cette demande se situe dans le domaine de la justice climatique. Les méthodes proposées pour atteindre les objectifs sont appropriées. Les activités proposées sont réalistes en fonction du temps et du budget disponibles.

La participation des populations ciblées est prévue aux différentes étapes du projet (entrevues individuelles et groupe de discussion). Une démarche du co-design centré sur l'expérience (CDCE) sera employée pour l'objectif 3 qui prévoit une collaboration entre les personnes utilisatrices de services, les prestataires de services et les «designers».

Les femmes en période périnatale sont les personnes visées par ce projet car elles constituent souvent le pont entre le réseau de la santé et les autres membres de leur famille. On veut développer des outils adaptés pour cette clientèle.

Faiblesses

Il manque de l'information sur les difficultés risquant de survenir durant la recherche et les solutions envisagées pour limiter leur impact.

2. Candidats

Forces

Globalement, les membres de l'équipe de recherche démontrent une solide expérience dans le domaine étudié (causes des migrations, santé des femmes migrantes, accès aux services de santé) et les méthodes de

Review Type/Type d'évaluation: Committee Member 4/Membre de comité 4
Name of Applicant/Nom du chercheur: Gagnon, Marie-Pierre
Application No./Numéro de demande: 528380
Agency/Agence: CIHR/IRSC
Competition/Concours: 2024-09-12 Catalyst Grant: Community Based Research in Climate Change Priority Areas/Subvention catalyseur : Recherche communautaire dans domaines prioritaires liés au changement climatique
Committee/Comité: Community Based Research in Climate Change Priority Areas/Recherche communautaire dans les domaines prioritaires du changement climatique
Title/Titre: Santé des familles immigrantes dans un contexte de justice climatique et de justice sociale (SI-JUSTE)

Assessment/Évaluation:

recherche proposées.

Faiblesses

Aucune faiblesse identifiée.

3. Impact de la recherche

Forces

La proposition de recherche porte sur un besoin important, soit l'impact de la migration environnementale sur la santé des femmes migrantes et de leur famille. L'accès aux services de santé est un enjeu important pour les nouveaux arrivants, en particulier pour les femmes dans la période périnatale, car leur prise en charge ne peut pas être retardée de plusieurs mois.

L'organisme impliqué offre des services à la clientèle visée. Il pourra contribuer au recrutement des participants, au co-design des outils et à la diffusion des résultats. Le projet s'appuie sur une collaboration bien établi avec la communauté visée et d'autres travaux de recherche pourront en découler.

Faiblesses

Le principal organisme impliqué dans le projet est le Service de référence en périnatalité pour les femmes immigrantes de Québec (SRPFIQ). Il serait utile d'identifier d'autres organismes œuvrant dans la région de Québec qui pourraient contribuer au projet.

Recommandation budgétaire

Budget approprié

Note : le budget est bien détaillé et toutes les dépenses sont justifiées.
